# Alpha-Synuclein: The Spark That Flames Dopaminergic Neurons, In Vitro and In Vivo Evidence

**DOI:** 10.3390/ijms23179864

**Published:** 2022-08-30

**Authors:** Alexandre Henriques, Laura Rouvière, Elodie Giorla, Clémence Farrugia, Bilal El Waly, Philippe Poindron, Noëlle Callizot

**Affiliations:** 1 Neuro-Sys, In Vitro Pharmacology Department, 13120 Gardanne, France; 2 Neuro-Sys Vivo, 13120 Gardanne, France

**Keywords:** Parkinson’s disease, α-synuclein, mitochondrial dysfunction, GBA, spreading

## Abstract

Mitochondria, α-syn fibrils and the endo-lysosomal system are key players in the pathophysiology of Parkinson’s disease. The toxicity of α-syn is amplified by cell-to-cell transmission and aggregation of endogenous species in newly invaded neurons. Toxicity of α-syn PFF was investigated using primary cultures of dopaminergic neurons or on aged mice after infusion in the SNpc and combined with mild inhibition of GBA. In primary dopaminergic neurons, application of α-syn PFF induced a progressive cytotoxicity associated with mitochondrial dysfunction, oxidative stress, and accumulation of lysosomes suggesting that exogenous α-syn reached the lysosome (from the endosome). Counteracting the α-syn endocytosis with a clathrin inhibitor, dopaminergic neuron degeneration was prevented. In vivo, α-syn PFF induced progressive neurodegeneration of dopaminergic neurons associated with motor deficits. Histology revealed progressive aggregation of α-syn and microglial activation and accounted for the seeding role of α-syn, injection of which acted as a spark suggesting a triggering of cell-to-cell toxicity. We showed for the first time that a localized SNpc α-syn administration combined with a slight lysosomal deficiency and aging triggered a progressive lesion. The cellular and animal models described could help in the understanding of the human disease and might contribute to the development of new therapies.

## 1. Introduction

Parkinson’s disease is the second most common neurodegenerative disorder, after Alzheimer’s Disease, and the most common movement disorder [1]. The pathology is characterized by motor symptoms including tremor, rigidity, bradykinesia, postural instability, gait and balance impairment and non-motor symptoms (cognitive decline, behavioral sleep disorder, constipation…) [2]. Additionally, its hallmark feature is the accumulation of α-synuclein (α-syn) protein forming cytoplasmic inclusions, known as Lewy bodies.

Parkinson’s disease is characterized by a gradual loss and degeneration of dopaminergic neurons in the *substantia nigra pars compacta* (SNpc). Its etiology is associated with several risk factors (environmental factors) and family history) [3].

The pathophysiological causes include mitochondrial dysfunctions, disruption of endo-lysosomes, endoplasmic reticulum (ER) stress and α-syn aggregation. The accumulation of α-syn into high molecular weight aggregates, induces cellular toxicity and may be one of the major contributors to pathogenesis in Parkinson’s disease [4]. The increased cellular toxic burden caused by aggregated α-syn may arise from overexpression of the protein, genetic multiplication, or a defect in the normal protein clearance mechanisms such as lysosomal autophagy [5].

α-syn is a structural protein particularly abundant in Tyrosine hydroxylase (TH)-expressing neurons where it is mainly located at the presynaptic terminal [6,7]. It is involved in synaptic activity through regulation of vesicle docking, fusion, and neurotransmitter release [8]. α-syn regulates the synthesis, storage, and release of dopamine (DA). It can interact with the components of the SNAP (Soluble NSF Attachment Protein) Receptor (SNARE) complex in the presynapse [9], but also interacts with the mitochondria and ER lipid membranes. Efforts to decipher the pathological role of α-syn depend on how the precise nature of the toxic protein species is defined. This is extremely challenging because during the aggregation process, α-syn adopts multiple conformations that exist simultaneously in a dynamic equilibrium between monomeric, oligomeric, and higher order aggregated states [10]. Indeed, it was recently shown that α-syn fibrils can interconvert into toxic oligomers, with these fibrils acting as a form of damaging species [11]. In fact, α-syn has been appropriately defined as a protein-chameleon due to its significant conformational plasticity; it may, under small thermal fluctuations, acquire different conformations that range from distinct secondary structure elements to a wide variety of high molecular weight assemblies [12].

In addition to the cellular toxicity, evidence of cell-to-cell transmission of pathological α-syn in interconnected brain regions suggests a “prion-like” mechanism of spread, causing toxicity and aggregation of endogenous species in newly invaded host cells [13].

Evidence showing that α-syn can transfer from a donor cell to a recipient cell was demonstrated in vitro [14] and in vivo [15], confirming the seminal findings of α-syn transfer from host to graft [16]. α-syn aggregates can be taken up by neurons via binding to the cell surface, or interaction with receptors such as cellular Prion Protein (PrPc) receptor, and this has been shown to mediate the internalization of α-syn [17]. Once inside the neuron, aggregated α-syn has been shown to be directed to the lysosomal compartment and transported along the axon [18]. The intracellular aggregates can be transmitted to neighboring cells via several possible routes: exported extracellularly in exosomes [19], or simply released after the neuronal death. This prion-like propagation of aggregated α-syn was further demonstrated following intra-striatal inoculation of recombinant α-syn pre-formed fibrils (PFF) in wild type mice.

The propagation of α-syn has also been demonstrated from the gut to the brain by injections of PFFs in the gut [20]. In addition to its ability to aggregate and propagate, α-syn can seed the formation of new aggregates. Many studies showed that biotinylated α-syn exogenous fibrils colocalize with newly formed endogenous α-syn aggregates [21].

Here we study the toxic events induced by a mixture of human oligomers/protofibrils of α-syn, in primary rat embryonic dopaminergic neurons in culture, using a standardized preparation representing different states of α-syn. We hypothesize that these species all existe in the Parkinson’s brain (at various levels) and that they participate to different extents to the physiological and pathological roles of α-syn. In this study, we worked on primary cultures of mesencephalic neurons, composed of 70% neurons (mainly GABAergic), 8% of which were dopaminergic neurons [22,23]. In order to specifically address these low contents of dopaminergic neurons (Tyrosine-hydroxylase-expressing neurons), we favored immunostaining rather than global investigation techniques (such as WB or qPCR) involving whole cell pellets.

We describe the early events leading to mitochondrial impairment, clearance deficits and finally TH-expressing neuron death. In addition, we sensibilized (at nontoxic concentrations) the neurons with a lysosomal glucocerebrosidase (GCase) inhibitor toxin ([conduritol-B-epoxide CBE]) in order to increase the α-syn lysosomal clearance deficit (also found in some genetic cases of Parkinson’s disease (Glucocerebrosidase (GBA1) gene mutation)). We followed the kinetics of neuronal death and proved the importance of the lysosomal function in α-syn clearance and toxicity. Finally, to probe the pathophysiological pertinence of our cellular findings, we extrapolated their conclusions to the whole animal, using aged mice (18-month-old). α-syn preparation was bilaterally injected into the SNpc. The chronic and progressive neuronal toxicity of α-syn (neuronal loss) was studied up to 2 months after α-syn infusion. The mitochondrial deficiency, the α-syn accumulation and the deficit of clearance were quantified over the time period. These events were associated with a massive and progressive central inflammation. We also proved that injection of α-syn induced behavioral deficits (motor coordination) that increased throughout time with the diffusion of the lesion.

In conclusion, we showed for the first time that a moderate α-syn administration in the SNpc, combined with a slight lysosomal GBA deficiency and advanced age, was sufficient to trigger a progressive lesion increasing over the time.

## 2. Results

### 2.1. α-Synuclein Preparation Induced Toxicity on TH Positive Neurons

The involvement of toxic forms of α-syn is well documented in Parkinson’s disease. Recent discoveries of biologically active α-syn oligomers/multimers (fibrils, protofibrils and aggregated) suggests a fine border between its physiological and pathological roles [24]. During the aggregation process, α-syn adopts multiple conformations that exist simultaneously in a dynamic equilibrium between monomeric, oligomeric and higher order aggregated states.

**Figure 1:**
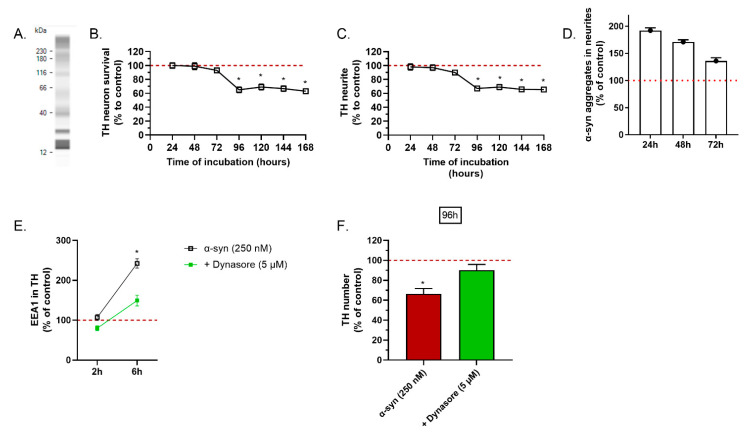
Effects of the different timing of α-syn incubation and dynasore on TH-expressing neurons and endocytosis. (**A**) WES analysis of α-syn preparation: virtual blot of the preparation composition. (**B**) Total number of TH(+) neurons (control = 23 ± 5), (**C**) length of TH(+) neurite network (control = 1261 µm ± 7), (**D**) area of cytoplasmic α-syn in TH neurons (control = 24 µm^2^ ± 5) in TH neurons after treatment with α-syn were studied after different incubation durations. (**E**) Expression of EEA1 marker in TH neurons (control = 204 µm^2^ ± 6) were studied 2 h and 6 h after application of dynasore and (**F**) survival of TH(+) neurons 96 h after (control = 114 ± 6). All values were expressed as mean ± SEM; *, *p* < 0.05 with One-way ANOVA followed by Fishers’ test.

The preparation of α-syn used in this study (for the in vitro and the in vivo studies) contained the different forms of the peptide. Indeed, we have detected monomeric and dimeric species, but also larger species compatible with oligomers and fibrils, at 40 kDa, 110 kDa and higher than 230 kDa (Figure 1A).

The application of the α-syn preparation on the mesencephalic cultures induced a significant loss of TH-dopaminergic neurons after 96 h of treatment (Figure 1B). The loss of neurons was concomitant with the loss of the neurite network (Figure 1C). This kinetic of toxicity suggests a progressive and slow toxicity. In Parkinson’s disease, evidence suggests that toxic species of α-syn propagate from cell-to-cell via endocytosis. Interestingly, we noted a pathological accumulation of α-syn aggregates in the neurites of TH(+) neurons 24 h after application of α-syn. This accumulation decreased over the time and was correlated with the loss of the neurite network (Figure 1D).

We hypothesized that the toxicity of α-syn was driven by an initial step of endocytosis. To test this hypothesis, we aimed to prevent cell entry of α-syn by blocking endocytosis with dynasore, an inhibitor of clathrin-mediated endocytosis. Dynasore was applied on the primary culture of mesencephalic cells 1 h before α-syn. First, we noted a clear increase in EEA1, a marker of endocytosis, in dopaminergic neurons, 6 h after application of α-syn. The α-syn-mediated activation of endocytosis was prevented by dynasore (Figure 1E). Most importantly, the chronic toxicity of α-syn on dopaminergic neurons was significantly reduced in culture treated with dynasore (Figure 1F).

**Figure 2:**
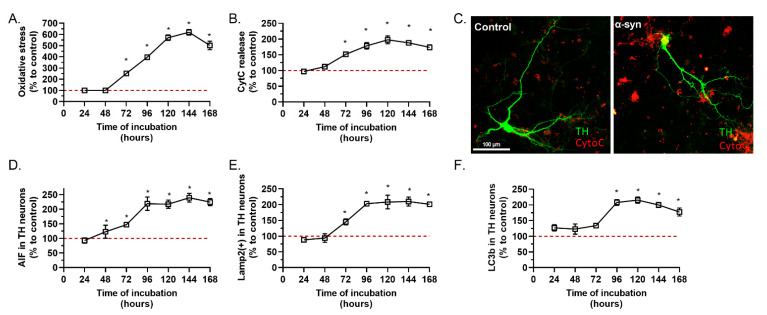
Effects of the different timing of α-syn incubation on mitochondrial stress and phagocytosis. (**A**) Total area of ROS signal (control = 22 ± 4) and (**B**) area of CytC (control = 58 µm^2^ ± 4) in TH(+) after intoxication with α-syn were studied after different incubation durations. (**C**) Representative pictures of TH neurons, control (left panel) and α-syn (right panel) for CytoC. (**D**) AIF (control = 42 ± 5) in TH(+) as well as (**E**) Lamp2(+) vesicles (control = 46 ± 3) and (**F**) area of LC3b(+) vesicles (control = 29 µm^2^ ± 3) after application of α-syn were also studied. All values were expressed as mean ± SEM; *, *p* < 0.05 with Two-way ANOVA followed by Fisher’s LSD test.

Mitochondrial dysfunction plays a central role in the mechanism of neurodegeneration in Parkinson’s disease. α-syn has been shown to be able to interact with mitochondrial proteins, such as TOM20 (translocase of the outer membrane 20), and to block mitochondrial protein import, induce complex I dysfunction and mitochondrial membrane potential dissipation, leading to the impairment of mitochondrial Ca^2+^ handling, generation of ROS and enhanced CytC release [25,26]. Application of α-syn on the primary culture of mesencephalic cells induced clear oxidative stress (Figure 2A), release of CytC (Figure 2B,C), a known effector of apoptosis, which led to the activation of caspase 3 (Supplementary Figure S3) and release of apoptosis inducer factor (AIF) (Figure 2D). Interestingly, the first significant generation of oxidative stress was observed 72 h after injury, one day before the loss of TH(+) neurons. Oxidative stress continued to increase, up to 144 h after the initial application of α-syn (Figure 2A,B,D).

Lysosomal pathway dysfunctions are characteristic of Parkinson’s disease. α-syn is degraded by the autophagy-lysosomal pathway and interacts with lysosomal proteins [27]. The application of α-syn in our primary culture induced a clear accumulation of lysosomes, after 72 h (Figure 2E,F). Interestingly, the accumulation of lysosomes parallels oxidative stress suggesting a temporal link between lysosomal and mitochondrial stress. We have also noted a significant accumulation of LC3(+) autophagosomes in the cytoplasm of TH(+) neurons, at 96 h after α-syn application (Figure 2F).

Altogether, these results indicate that the toxicity of α-syn species for TH(+) neurons is dependent on functional endocytosis, and triggers mitochondrial and lysosomal impairments.

### 2.2. Reduced Activity of Lysosomal GBA Exacerbates the Toxicity of Alpha-Synuclein on Primary Dopaminergic Neurons

Genetic mutations on GBA, a lysosomal enzyme involved in the degradation pathway of glycosphingolipids, impairs its trafficking and reduced total activity in the lysosome. Gene mutations on GBA are a known risk factor for developing Parkinson’s disease and other synucleinopathies [28]. Many reports showed that a decreased enzymatic activity of the lysosomal GCase has been measured in the SN of sporadic and familial parkinsonian patients, and correlated with the accumulation of α-syn. We hypothesized that impairing GBA activity with an inhibitor would exacerbate the toxicity of α-syn on TH(+) neurons.

**Figure 3:**
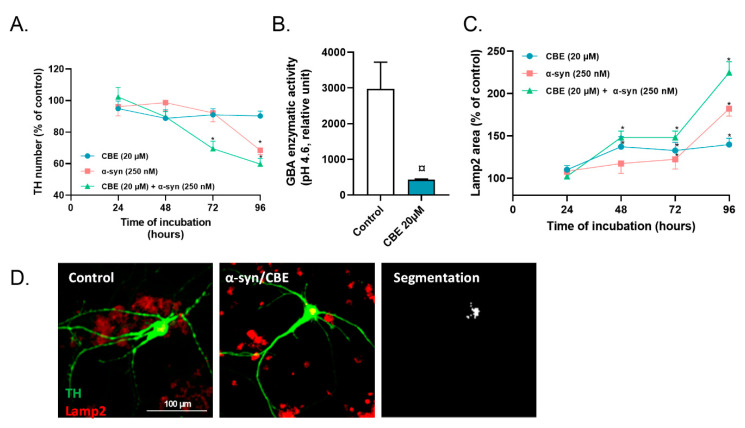
Progressive loss of TH(+) neurons after application of α-syn or α-syn + CBE. (**A**) Number of TH(+) neurons (control = 23 ± 5) after different incubation times with CBE and α-syn, (**B**) enzymatic GBA activity following CBE intoxication and (**C**) lysosomal impairments (control = 46 ± 3) after different incubation times. (**D**) Representative pictures of Lamp2 staining (red) in TH neurons (green) and the computer-assisted segmentation resulting from data processing (white): control condition (left panel), α-syn condition (middle panel) and segmentation (right panel). All values are expressed as mean ± SEM; *, *p* < 0.05 with One-way ANOVA followed by Fisher’s LSD test; or ¤ *p* < 0.05 with Mann-Whitney test.

We applied CBE, a covalent inhibitor of GBA, on the primary culture of dopaminergic neurons, at 20 µmol/L. At this concentration, application of CBE is not associated with a significant loss of dopaminergic neurons (Figure 3A) but significantly reduced the enzymatic activity of GBA and promoted an accumulation of lysosomes in dopaminergic neurons (Figure 3B,C).

A combined application of α-syn and CBE resulted in a loss of dopaminergic neurons after 48 h, whilst without CBE, the first loss of dopaminergic neurons mediated by α-syn was observed after 96 h (Figure 3A). Interestingly, the combined application of CBE and α-syn resulted in a massive accumulation of lysosomes in dopaminergic neurons, suggesting massive lysosomal burden. (Figure 3C,D).

### 2.3. Intra-Nigral Injection of Alpha-Synuclein and Chronic Inhibition of GBA Induce Motor Dysfunction and Loss of Dopaminergic Neurons in Aged Mice

Parkinson’s disease is a neurodegenerative disease associated with age and characterized by the loss of dopaminergic neurons in the SNpc, lysosomal dysfunctions and as a synucleinopathy [29]. Previous reports indicate an absence of a clear phenotype after injections of α-syn in the brain of rodents. In addition, our in vitro results, described above, clearly support a negative synergy between α-syn and lysosomal dysfunction. Therefore, we aimed to investigate the effect of α-syn injections in the SNpc of 18-month-old mice, combined with a chronic inhibition of GBA. Bilateral stereotaxic injections of α-syn were made on mice at 18 months of age and CBE was given 3 times per week (on the overall period of the study), at 50 mg/kg, by intra-peritoneal injections.

**Figure 4:**
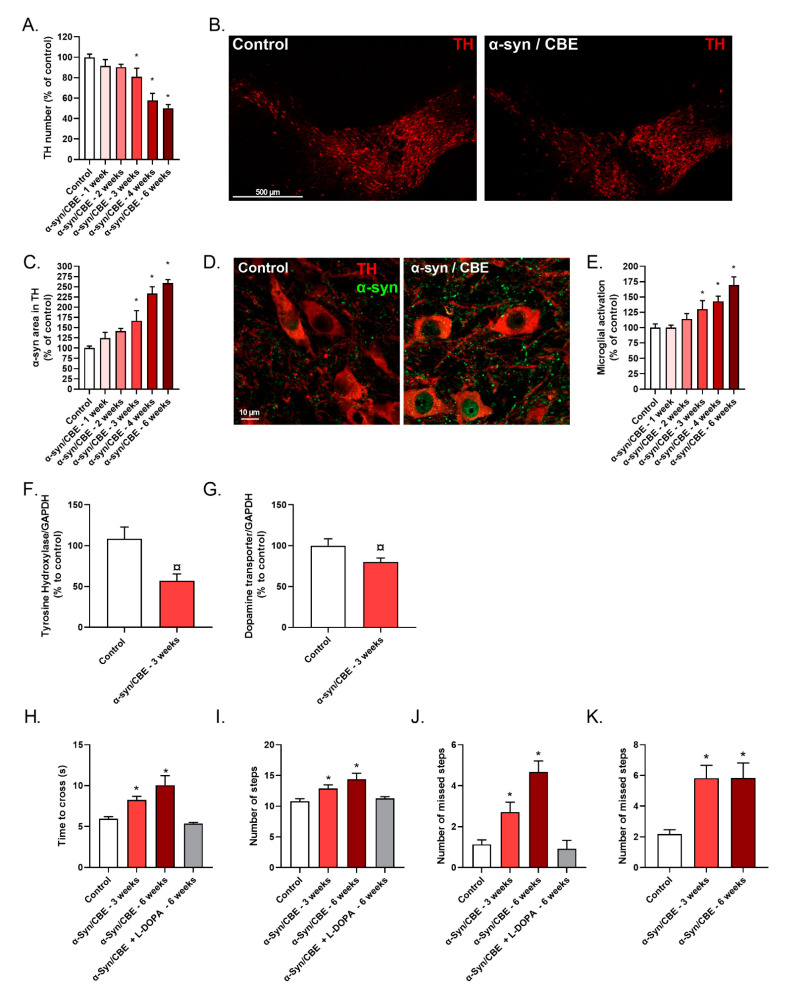
Effects of α-syn stereotaxic injections over the time in the *substantia nigra* of aged mice and on motor coordination in aged mice. (**A**) Number of TH(+) neurons (control = 79 ± 6) and (**B**) representative pictures of SNpc in control mice (left panel) and α-syn mice (right panel) stained for TH (+) neurons (red). (**C**) Area of cytoplasmic α-syn in TH(+) neurons (control = 233 µm^2^ ± 18) in TH(+) after α-syn injections were studied. (**D**) Representative pictures of SNpc in control mice (left panel) and α-syn mice (right panel) stained for TH(+) neurons (red) and for α-syn (green). (**E**) Microglial activation assessed by the number of IBA1(+) microglia (control = 35 ± 15) in TH(+) after α-syn injections. (**F**) TH and (**G**) DAT protein level in SNpc of aged mice. Mice performance on motor behavioral test were also studied: time to cross the bar (**H**), number of steps in the bar test (**I**), number of times the mice slip off the bar (**J**) and the number of missed paws (i.e when the mice slip off the grid with one paw) (**K**), compared to control aged mice. Results are expressed as a percentage of control condition as mean ± SEM (**A**–**E**) or as mean ± SEM (**G**–**J**); ¤, *p* < 0.05 with One-way ANOVA followed by Fisher’s test. *, *p* < 0.05 with non-parametric Mann–Whitney’s test.

In our model, 3 weeks after intra-nigral α-syn injections and GBA inhibition (α-syn/CBE), a progressive loss of TH(+) neurons was observed (Figure 4A,B). This progressive loss of neurons was associated with an α-syn aggregation (significant) in the TH(+) neurons, starting 3 weeks after α-syn injections and progressively increasing up to 6 weeks post-surgery (Figure 4C,D). In addition, activation of microglial cells (also significant) was also observed 3 weeks after α-syn injections (Figure 4E), when compared to control group. These results were also supported by protein analysis, showing, in the SNpc of α-syn/CBE mice, a significant decrease of TH and DAT protein levels, 3 weeks post-surgery (Figure 4F,G). Matching the in vitro experiments, we confirmed in vivo, a slow and progressive α-syn toxicity.

These histological observations were well correlated with the motor dysfunctions of the animals. In the bar test, we found, on week 3 after the surgery, that the α-syn/CBE mice crossed the bar more slowly as compared to the control mice (Figure 4H). α-syn/CBE mice also needed more steps to reach the platform (Figure 4I) and presented a higher number of slips compared to control mice (Figure 4J). These motor dysfunctions were still observed 6 weeks after surgery, but were not exacerbated. Interestingly, after an acute administration of L-Dopa, a DA precursor used as reference treatment in Parkinson’s disease, motor impairments were attenuated. Indeed, mice managed to cross the bar as quickly as the control mice (Figure 4H). Moreover, the number of steps and slips were also lower after L-Dopa injection, back to the control mice level (Figure 4I,J).

In the grid walking test, 3 weeks after the surgery, the α-syn/CBE mice showed an increased number of missed steps compared to the control mice (Figure 4K). Similar observation was seen 6 weeks after the surgery, supporting the fact of motor deficit maintained over the time.

**Figure 5:**
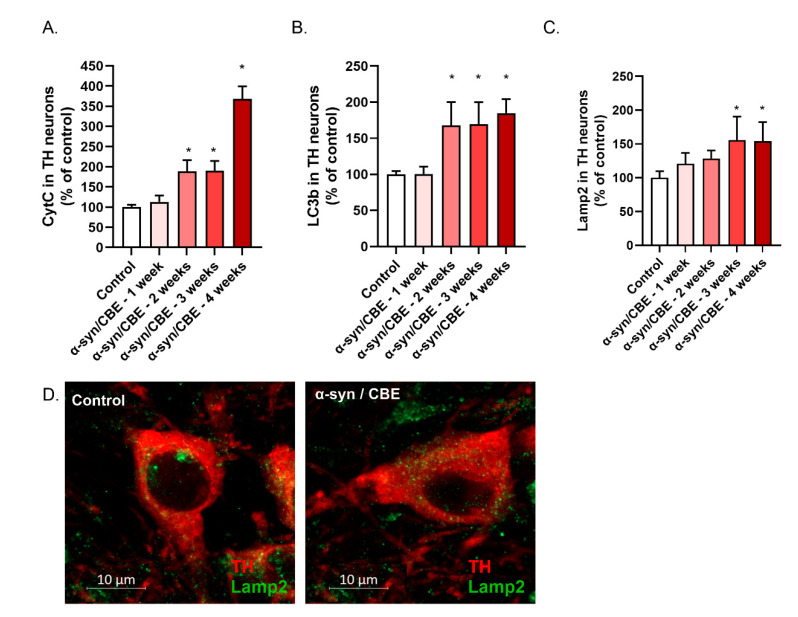
α-syn/CBE mice present with progressive mitochondrial stress and autolysosomal stress in the *substantia nigra*. Total area of CytC (control = 1150 µm^2^ ± 10) (**A**), as well as area of LC3b (control = 7 µm^2^ ± 9) (**B**) and Lamp2 (control = 2027 µm^2^ ± 13) (**C**) in TH(+) after stereotaxic injection of α-syn were studied. (**D**) Representative pictures of TH(+) control (left panel) and α-syn (right panel) for Lamp2 (green). All values are expressed as mean ± SEM; *, *p* < 0.05 with one-way ANOVA followed by Fisher’s LSD test.

As mentioned in our in vitro experimental section, the mitochondrial dysfunction plays a central role in the mechanism of neurodegeneration in Parkinson’s disease. Bilateral stereotaxic injections of α-syn in the SNpc of aged mice and chronic GBA inhibition induced obvious release of CytC, already 2 weeks after surgery (Figure 5A). This mitochondrial dysfunction was associated with a large increase of LC3b autophagic vesicles (Figure 5B) and of LAMP2 lysosomal vesicles, that reached a plateau at 3 weeks (Figure 5C,D).

These mitochondrial and lysosomal defects were observed one week before the loss of TH(+) neurons.

## 3. Discussion

The pathophysiology of Parkinson’s disease still remains unclear. However, new observations have highlighted the key roles of several protagonists in the disease progression such as the mitochondria (in dopaminergic neurons in which the energy demand is very high), α-syn, the endo-lysosomal system (α-syn, as a presynaptic protein that physiologically helps to regulate synaptic vesicle transportation and endocytosis). Furthermore, some studies have demonstrated that α-syn exhibits a “prionoid” behavior, implying that α-syn propagates in a prion-like manner [24,30].

The PFF injection induced the formation of α-syn-positive cytoplasmic inclusions in neurons of the striatum as well as interconnected regions, such as the cortex, which are associated with a loss of dopaminergic neurons in the SN which can lead to motor dysfunctions [31]. A similar injection of soluble α-syn had no effect [32]. PFF or Lewis Body-enriched fractions purified from Parkinson brains containing pathological aggregated α-syn also induced a progressive synucleinopathy and nigrostriatal neurodegeneration after intracerebral injection into susceptible transgenic mice [33], or in non-human primate models [34]. These pathogenic effects were abolished when injections were performed into mice lacking α-syn expression, or when inoculates were experimentally deprived of α-syn.

Here, we showed, on primary dopaminergic neurons, that application of α-syn preparation (containing oligomers and PFF) induced a progressive cytotoxicity with mitochondrial dysfunction and oxidative stress.

Counteracting the α-syn endocytosis (using a clathrin inhibitor), the DA neuron degeneration was massively prevented. Using some markers of endocytosis, we also proved that α-syn was able to be internalized via a dynamin-dependent, clathrin-mediated endocytosis in the DA neurons a few hours after the application (as previously shown on neuroblastoma and hippocampal neurons). Other pathways could be involved in the intracellular α-syn transmission, for example caveolin (increased with age) overexpression in neurons was shown to increase the cell-to-cell transmission of α-syn [35].

Here we proved that after 48 h–72 h mitochondrial dysfunction (AIF, CytC, ROS) occurred. Concomitantly, increased lysosomal marker (LAMP2a) was observed (72 h) suggesting that exogenous α-syn reached the lysosome (from the endosome) for clearance to reduce the α-syn level [18]. Interestingly, 96 h after the α-syn application, an increase of LC3II levels was observed, in favor of autophagolysosomal burden. Aggregated α-syn (A53T and A30P mutations) has been reported to exhibit a strong binding to LAMP2a that could disturb the entire cellular homeostasis [24].

As mentioned above, in parallel to the protein clearance impairment, some mitochondrial dysfunctions were observed, that could be explained by a direct action of α-syn with the mitochondria. Indeed, it has been proved that α-syn (especially A53T) directly binds to the mitochondrial outer membrane and enhances respiratory dysfunction [25,36], AIF and CytC leakages and ROS production. These dysfunctions could also be linked to the autophagolysosome burden [37].

Surprisingly, only a limited proportion of the neurons died after α-syn application. The surviving neurons (60%) showed dysfunctions (mitochondrial and/or lysosomal impairments) over time but they did not die, proving that some populations of dopaminergic neurons are more sensitive than others, as shown by Decressac et al. [38]. In addition, some authors showed that deficit in protein clearance can induce susceptibility to α-syn toxicity [38].

Moreover, in Parkinson’s disease, it is well known that GBA1 mutation, reducing its lysosomal activity, is a high risk factor to develop Parkinson’s disease [39,40].

In fact, inhibition of GBA leads to a dramatic increase in α-syn pathology when this pathology is initiated with misfolded α-syn seeds [39]. This was confirmed with our cellular results; the application of CBE (at sub-toxic concentrations) accelerated the aggregated α-syn defect and toxicity. This confirms that GBA loss of activity is an underlying disease susceptibility and does not trigger the pathology [40].

In the whole animal, the α-syn preparation induced similar features observed on primary cultures. Infused in the SNpc, a progressive dopaminergic neuronal loss was observed over time, associated with a massive α-syn accumulation. Interestingly the lesion area increased with time suggesting a cell-to-cell toxicity triggered by the infusion of α-syn. A cell-to-cell propagation pathway implies that α-syn is released from cells, taken up by neighboring cells and stimulates the aggregation of endogenous α-syn within recipient cells, probably serving as a “seed” for further aggregation processes.

As we showed, with other authors, GBA inhibition does not necessarily increase the total α-syn levels or lead to de novo aggregation of α-syn [40,41]. Our results were in agreement with these observations. Indeed, in the cell or animal model, we proved that administration of CBE alone (at the concentrations used in combination) did not lead to any toxic α-syn accumulation. By contrast, when the pathology was already triggered (with α-syn application alone), GBA1 inhibition sped up the kinetics of DA neuron death in vitro and in vivo (Supplementary Figure S4). Our findings are in line with a sensitization or an increase in the vulnerability of the DA neurons. In addition, reduced GBA activity leads to accumulation of glucosylceramide (GlcCer) and glucosylsphingosine (GlcSph) [42]. The accumulation of these substrates can impair lysosomal function [43], possibly leading to reduced degradation of pathogenic α-syn. Furthermore, when the lysosome is overburdened, vesicle contents may be released into the extracellular space [44]. These released vesicles may contain GlcCer and GlcSph as well as pathogenic α-syn, enhancing the cell-to-cell spread of α-syn. In this cell-to cell propagation, we cannot exclude the role of the mitochondria. Some authors showed mitochondria-mediated α-syn intercellular transfer via tunnelling nanotubes [45].

In our animal model we observed a progressive neurodegeneration in SNpc associated with a loss of TH and DAT content and motor deficits. The aggregation of α-syn was progressive and accounted for the seeding role of primary injection of α-syn which acted as a spark. This hypothesis was supported by in vitro observations of mitochondrial dysfunction, lysosomal overload (supported by GBA inhibition) and finally aggregation of α-syn and its propagation to the surrounding neurons. Interestingly, this degeneration was also associated with microglial activation. We observed an increase in Iba1 expressing cells in striatum and SNpc, hallmark of active microglia cells. Microglia, the main resident immune cells in the brain, phagocytose dead cells and help to clear misfolded α-syn aggregates in Parkinson’s disease [46]. It has been shown that microglia are also actively involved in the process of cell-to-cell transmission of α-syn through the release of exosomes [47]. Guo et al., 2020 [47] showed that α-syn aggregated microglial exosomes could induce nigrostriatal degeneration. In addition, the multiple roles of the microglia in the cell-to-cell transmission was well described by George et al., 2019 [48] showing that non-activated microglia decreased α-syn neuron-to-neuron transfer; however, when activated using LPS, neuron-to-neuron transfer was increased. Thus, in our system we cannot exclude the role of microglial cells in the propagation of the pathology. We observed a progressive increase of the activation starting 2 weeks after the α-syn infusion.

It is noteworthy that the drop of TH and DAT, the massive inflammation and the loss of dopaminergic neurons lead to motor coordination deficits. They were significant 3 weeks after the infusion when 40% of the DA neurons were lost. These motor coordination deficits were reversed with an acute administration of L-Dopa.

Overall, this animal model presents many common features with the human pathology—the advanced age of the animals, the progressive DA degeneration associated with a α-syn aggregation and a neuroinflammation increasing over time. The neuronal death leads to DA depletion and finally to motor coordination deficits. In addition, we evidence in vitro and confirm in the whole animal important mechanisms involved in the progression of the pathology: the central role of the mitochondria, the impairment of lysosomal function and the seeding role of α-syn in the cell-to-cell propagation. The sensitivity of some populations of DA neurons as well as the advanced age of the animals seemed to be important components in the disease progression.

In conclusion, we showed for the first time that a moderate α-syn administration in the SNpc combined with a slight lysosomal GBA deficiency and advanced age was sufficient to trigger a progressive lesion increasing over time. The features of the sequential cellular pathway of death and the animal lesion showed similarities to the human pathologies. We assume that the cellular and animal models described above could be helpful in the understanding of the human disease and might contribute to the development and validation of new therapies.

## 4. Materials and Methods

Animals were housed for acclimatation for 1 week in Neuro-Sys facilities and were maintained in a reversed 12 h light–dark cycle. The animals were group-housed (2–4 mice per cage, 2 rats per cage) in ventilated cages, and maintained in a room with controlled temperature (21–22 °C) and hygrometry (40–60%) and with ad libitum food and water available.

### 4.1. Primary Culture of Mesencephalic Neurons

Pregnant female rats of 15 days gestation (Rats Wistar; Janvier Labs, Le Genest-Saint-Isle, France) were killed using a deep anesthesia with CO_2_ chamber followed by cervical dislocation.

Rat mesencephalic neurons were prepared and cultured as described in Callizot et al. (2019) [23]. Briefly, the midbrains were dissected under a stereo zoom binocular microscope. The embryonic midbrains were collected from 15-day old rat embryos and placed in ice-cold L15 medium of Leibovitz (L15, Pan Biotech, Aidenbach, Germany) containing 2% of penicillin (10,000 U/mL) and streptomycin (10 mg/mL) solution (PS, Pan Biotech, Aidenbach, Germany) and 1% of bovine serum albumin (BSA, Pan Biotech, Aidenbach, Germany). The ventral portion of the mesencephalic flexure, a region of the developing brain rich in dopaminergic neurons, was used for the cell preparations. The midbrains were dissociated by trypsinization for 20 min at 37 °C (Trysin 0.05% EDTA 0.02%, Pan Biotech, Aidenbach, Germany). The reaction was stopped by the addition of Dulbecco’s modified Eagle’s medium (DMEM, Pan Biotech, Aidenbach, Germany) containing 0.1 mg/mL of DNase I grade II (Pan Biotech, Aidenbach, Germany) and 10% of fetal calf serum (FCS, Thermo Fisher Scientific, Cergy Pontoise, France). Cells were then mechanically dissociated by 3 passages through a 10 mL pipette. They were then centrifuged at 180× *g* for 10 min at +4 °C on a layer of BSA (3.5%) in L15. The supernatant was discarded and the cell pellets were re-suspended in a defined culture medium consisting of Neurobasal medium (Thermo Fisher ScientificInvitrogen, Cergy Pontoise, France) with the addition of 2% of B27 complement (Invitrogen), 2 mmol/L of L-glutamine (Pan Biotech, Aidenbach, Germany), 2% of PS solution, 10 ng/mL of Brain-derived neurotrophic factor (BDNF, Pan Biotech, Aidenbach, Germany) and 1 ng/ mL of glial-derived neurotrophic factor (Pan Biotech, Aidenbach, Germany). Viable cells were counted in a Neubauer cytometer using the trypan blue exclusion test. The cells were seeded at a density of 40,000 cells/well in 96 well-plates pre-coated with poly-L-lysine (Corning Biocoat, Le Pont de Claix, France) or 225,000 cells/well in 24 well-plates (Greiner, Courtaboeuf, France) coated with poly-L-lysine ([10 μg/mL, for 2 h], Sigma Aldrich, St. Louis, MO, USA) and maintained in an air (95%) plus CO_2_ (5%) humidified incubator, at 37 °C. Half of the medium was replaced every 2 days with fresh medium. The mesencephalic cell cultures were treated after 6 days of culture. The dopaminergic neurons were identified by immunostaining of TH. As mentioned by Visanji and colleagues [22], in our conditions, TH-expressing neurons represented 6% to 8% of the neuronal population, although under these conditions, a residual population of microglial cells remain, accounting for ~1% of all cells.

### 4.2. Treatment of Primary Mesencephalic Cultures

Neurons were injured with a preparation of human α-syn (R-peptide, Watkinsville, GA, USA), reconstituted in defined culture medium at 4 µM and slowly shaken at +37 °C for 3 days in darkness to induce oligomerization [49] (see Supplementary Figure S1 for α-syn preparation and content).

After 6 days of culture, the dopaminergic neurons were injured with α-syn (250 nmol/L) up to 168 h. Dynasore (Selleckchem, Houston, TX, USA), an inhibitor of clathrin-mediated endocytosis was applied for 2 h, 6 h or 96 h, from day 6 of culture, in presence of α-syn (250 nmol/L). Conduritol B epoxide (MedChem Express, Sollentuna, Sweden), an inhibitor of GBA, was applied alone (at 20 µmol/L, inducing around 50% decrease of GBA activity) or combined with α-syn (250 nmol/L), on day 6. The medium was removed and replaced with fresh medium containing or not α-syn (250 nmol/L), CBE or dynasore every other day.

### 4.3. Immunostaining and Automatized Image Analysis

At the end of the culture, the cells were washed with phosphate-buffered saline (PBS; Pan Biotech, Aidenbach, Germany) and fixed with a solution of 4% paraformaldehyde (PFA; Sigma Aldrich, St. Louis, MO, USA) in PBS, pH 7.3, for 20 min at room temperature (RT, 21 °C). They were washed twice again in PBS, and were then permeabilized and the non-specific sites were blocked using a solution of PBS containing 0.1% of saponin (Sigma Aldrich, St. Louis, MO, USA) and 1% of FCS, for 15 min at RT. All primary antibody (Ab) incubations were performed in PBS containing 1% of FCS, 0.1 mg/mL of saponin, for 2 h, at RT (21 °C). The cells were then washed with PBS containing 1% of FCS, 0.1% of saponin, and then incubated with the appropriate secondary Ab (goat anti-mouse, labeled with Alexa 488 or goat anti-rabbit, labeled with Alexa 568, Invitrogen), used at 5 μg/mL in PBS containing 1% of FCS and 0.1% of saponin, for 1 h at RT. The different Ab, and detailed procedures used in this study are listed in Table 1. Briefly, cultures were stained with antibodies anti-tyrosine hydroxylase (TH, to identify dopaminergic neurons), combined with antibodies anti-α-syn, anti-cytochrome C (marker of apoptosis), anti-EEA1 (marker of endosomes), anti-LC3b (marker of autophagosomes), or anti-Lamp2 (marker of lysosomes). Oxidative stress in dopaminergic neurons was evaluated with CellROX reagent (Thermo Fisher Scientific branch, Illkirch, France). CellROX reagent contains a probe that becomes fluorescent in contact of reactive oxygen species. After injury, the cell culture supernatant was removed, and live cells were exposed to CellROX reagent (marker of reactive oxygen species (ROS) production) for 30 min at 37 °C. The CellROX reagent is cell-penetrant and will become fluorescent once oxidized by ROS. Cells were fixed by a solution of 4% paraformaldehyde in PBS, pH = 7.3 for 20 min at room temperature and stained with an antibody anti-tyrosine hydroxylase. The area of ROS fluorescent probe in TH(+) neurons was determined by image analysis. Cell nuclei were counterstained with the fluorescent dye Hoechst (Sigma Aldrich).

Photomicrographs (20 pictures per well, at 10× magnification) were automatically acquired on ImageXpress^®^ microscopic system (Molecular Devices, San Jose, CA, USA). For each condition (6 culture wells), 20 fields per well (representing 80% of the total surface of the well) were automatically analyzed using MetaXpress^®^ software (Molecular Devices, San Jose, CA, USA). For the detection of fluorescence, the cell analyzer took into consideration cells presenting a signal equal to/or greater than/a threshold automatically fixed by observation of cultures treated only with secondary antibodies. This threshold is very low, since secondary antibodies did not label the cells. All wells were observed under exactly the same conditions, so as the objects detected by the cell analyzer belonged to the same class, rendering possible statistical comparisons. Read-outs included the number of TH positive neurons, length of the neurite network, and the area of specific markers in TH-positive cell bodies. The results were expressed in terms of percentage of neurons displaying the characteristic analyzed, as compared to control conditions.

### 4.4. Mice; Stereotaxic Injection of Alpha-Synuclein and Chronic Inhibition of GBA

C57BL6 mice (18 months old) were provided by Janvier Labs (Saint Berthevin, France). One week after their arrival, all mice were subjected to surgery and received either the vehicle (NaCl, 0.9%, CentraVet, Lapalisse, France) or the α-syn preparation (50 µmol/L final concentration, see Supplementary Figure S1 for α-syn preparation and content). Mice were anesthetized by isoflurane (4%, for induction, CentraVet, Lapalisse, France) in an induction chamber coupled with a face mask coupled to the isoflurane vaporizer and oxygen concentrator. Mice were later placed on the stereotaxic frame. Anesthesia was maintained by isoflurane (2%). The skull was exposed and holes were drilled to allowed stereotaxic injections.

α-syn preparation (50 µmol/L, 2.5 µL/injection site) or vehicle (NaCl 0.9%) were bilaterally injected with a Hamilton syringe (0.1 µL/min with an Elite Nanomite syringe pump) in the Substantia nigra pars compacta (SNpc) at the following coordinates: A-P, −0.3 cm; M-L, ±0.12 cm; D-V, −0.45 cm, according to the Paxinos and Franklin’s mouse brain atlas (Franklin, Keith B.J., and George Paxinos, 1997). SNpc was chosen as the site of injection in order to allow a direct contact between alpha-synuclein oligomer/fibrils and the cell body of dopaminergic neurons.

Depth of anesthesia and rectal temperature were checked every 5 min. After surgery, mice were allowed to recover for a week.

Chronic inhibition of GBA (50% of the total activity) was achieved with conduritol B epoxide (solubilized in NaCl 0.9%, 50 mg/kg, 3 times per week, i.p.) (Supplementary Figure S2). The injection volume was at 100 µL/10 g of body mass of the mouse.

### 4.5. Histology and Automatized Image Analysis

At the end of the experiments (1, 2, 3, 4 or 6 weeks after surgery), mice were deeply anesthetized and perfused with cold PBS (3 min), and cold PFA 4% in PBS (3 min). Brains were dissected and further fixed in PFA 4%, overnight at 4 °C. Then, brains were placed in 30% sucrose in Tris-phosphate saline (TBS) solution at 4 °C. Coronal sections, including the SNpc, of 40 µm-thickness were cut using a cryostat (4 sections per mouse, each 100 µm apart). Free-floating sections were incubated in TBS with 0.25% bovine serum albumin, 0.3% Triton X-100 and 1% goat serum, for 1 h at RT. This incubation blocked non-specific binding sites and permeabilized the tissues.

Four (*n* = 4) brain sections per animal were processed and incubated overnight at RT with selected Abs (See Table 2). Briefly, brain sections were stained with antibodies anti-TH, combined with antibodies anti-α-syn, anti-cytochrome C, anti-IBA1, anti-LC3b, or anti-Lamp2. These Abs were revealed with Alexa Fluor 488 anti-rabbit IgG, Alexa Fluor 568 anti-chicken IgG, at the dilution 1/500, incubated in TBS with 0.25% bovine serum albumin, 0.3% Triton X-100 and 1% goat serum.

Images were acquired with a confocal microscope LSM 900 with Zen software at 20× magnification using the same acquisition parameters (automatic acquisition). From images, the analyses were automatically performed by MetaXpress^®^ (Molecular Devices, San Jose, CA, USA). The following read-outs were measured in the SNpc: (a) the number of TH positive cells [TH(+)], (b) the aggregation of α-syn in TH-positive cells, (c) the cytochrome C (CytC) area in TH(+) cells, (d) the LC3b area in TH-positive cells, (e) the Lamp2 area in TH(+) cells and f) the number of Iba1-positive microglial cells.

### 4.6. Brain Protein Analysis

Mice were deeply anesthetized and perfused with cold PBS (3 min), brains were dissected and immediately frozen at −80 °C. Brain samples were lysed with a defined buffer lysis consisting of CelLyticMT reagent with 1% of protease and phosphatase inhibitor cocktail (60 μL per well). For each condition, the quantity of protein was determined using the micro kit BCA (Pierce, Thermofisher, Waltham, MA, USA). Briefly, lysates were diluted to 1/50 in PBS and mixed, in equal volume, with a micro BCA. After an incubation at 60 °C for 1 h, the quantity of proteins was measured at 562 nm with a spectrophotometer Nanovue (GE Healthcare, Chicago, IL, USA) and compared with the standard of Bovine Serum Albumin curve (BSA, Pierce, Thermofisher, Waltham, MA, USA).

All reagents (ref: SM-W004, except primary Abs) and secondary Abs (ref: DM-001 or DM-002) were provided by ProteinSimple^®^. They were prepared and used according to manufacturer’s recommendations for use on WES™ (ProteinSimple, San Jose, CA, USA). The run was performed according to recommendations. Capillaries, samples, Abs, and matrices were loaded inside the WES apparatus. The concentration of proteins was adapted for each protein and ranged between 0.2 and 1.5 mg/mL. The Simple Western was run with capillaries filled with separation matrix, stacking matrix and protein samples. Next, capillaries were incubated at RT for 2 h with the following primary Abs: (a) anti-TH (Sigma-Aldrich, D6944, 50 kDa) or (b) anti-DAT (dopamine transporter; Cell Signaling, 2507S, 80 kDa).

Capillaries were washed and then incubated with horse radish peroxidase-conjugated secondary Abs for 1 h, at RT. After removal of unbound secondary Ab, the capillaries were incubated, at RT, with the luminol-S/peroxide substrate and the chemiluminescent signal was recorded using the Charge-Coupled Device camera of WES™ with six different exposure times (30, 60, 120, 240, 480, and 960 s). Data analysis was performed using the Compass Software version 6.0.0 (ProteinSimple, San Jose, CA, USA) on WES™.

### 4.7. Evaluation of Motor Functions, Behavioral Analysis

All the behavioral sessions were conducted in a quiet room, and all the apparatus was visually isolated from the rest of the room to avoid any perturbance. Before each trial, the surface was wiped clean with 70% alcohol and dried. All the trials were video-recorded and replayed for analysis. Behavioral tests were performed on week 3 and week 6 after surgery.

Motor functions were evaluated on the grid walking test and on the fixed bar test. For the grid walking test, a horizontal square grid setup (30 cm × 50 cm) consisted of a wire mesh (openings 2 cm) and was mounted 100 cm above the floor. This test assesses motor coordination of the animals. On weeks 3 and 6 post-surgery, mice were placed on the middle of the grid, and allowed to freely walk for 1 min. Stepping errors (misplacement of the paws leading to a slip through the grid) were recorded. Mice were subjected to 3 sessions a day (inter trial interval, ITI 10 min), for 3 consecutive days. The number of errors is directly correlated to a motor deficit. The fixed bar test apparatus consisted of a horizontal bar (18 cm diameter and 65 cm length). Mice were placed at one end of the bar and were left to cross the bar with a maximal time of 60 s. Each mouse was habituated in two days of training and was tested on the third day. The test session consisted of 3 trials each (ITI: 10 min). The mean of the 3 trials was considered for analyses; the following readouts were recorded: (a) time to cross the bar, (b) the number of missed steps, and (c) the total number of steps.

### 4.8. Statistical Analysis

For the in vitro and in vivo experiments, the level of cytopathic effects was expressed in percentage of control conditions (no injury or no α-syn = 100%). All values were expressed as mean +/− SEM (standard error of the mean) of 6 wells (in vitro) or of 7 mice per group for histology and the protein analysis (WES) or 10–12 mice per group for motor functions (in vivo). For each control condition (in vitro or in vivo) on each graph, the raw values were mentioned to provide enough information about level of damage recorded.

Statistical analyses were done using one-way or two-way ANOVA followed (when allowed) by Fisher’s LSD test, or a Mann–Whitney test. *p* < 0.05 was considered significant. Graphs and statistical analyses on the different conditions were performed using GraphPad Prism software version 9 (GraphPad software Inc., La Jolla, CA, USA).

## Figures and Tables

**Table 1:** List of antibodies used in the present study for immunohistochemistry.

Antibody	Characteristic	Fixation	Dilution	Provider	Reference
Tyrosine hydroxylase (TH)	Mouse monoclonal	PFA	1/2000	Sigma	T1299-.2ML
alpha-synuclein (α-syn)	Rabbit polyclonal	PFA	1/200	Cell signaling	2642S
Early endosome antigen 1 (EEA1)	Mouse monoclonal	PFA	1/100	Abcam	Ab70521
Lysosome-associated membrane protein 2A (Lamp2)	Rabbit polyclonal	PFA	1/200	Abcam	Ab203224
Microtubule-associated protein light chain 3 beta (LC3B)	Rabbit polyclonal	PFA	1/100	Cell signaling	3868S
Reactive oxygen species (ROS)		On live cells	1/2000	Fisher	12648335
Allograft inflammatory factor-1 (AIF)	Mouse monoclonal	PFA	1/100	Santa Cruz biotechnology	SC-13116
Cytochrome C (CytC)	Rabbit polyclonal	PFA	1/100	Abcam	ab90529
Caspase 3	Rabbit polyclonal	PFA	1/100	cell signaling	9662

**Table 2:** List of antibodies used in the present study for immunohistochemistry.

Antibody	Characteristic	Fixation	Dilution	Provider	Reference
Tyrosine hydroxylase (TH)	Polyclonal, chicken	PFA	1/1000	Abcam	Ab76442
Alpha-synuclein (α-syn)	Polyclonal, rabbit	PFA	1/200	Cell Signaling	2642S
Ionized calcium-binding adaptator molecule 1 (IBA1)	Polyclonal, rabbit	PFA	1/500	Novus	NBP2-19019
Cytochrome C (CytC)	Polyclonal, rabbit	PFA	1/100	Abcam	Ab90529
Microtubule-associated protein light chain 3 beta (LC3b)	Monoclonal, rabbit	PFA	1/200	Cell Signaling	3868S
Lysosome-associated membrane protein 2A (Lamp2A)	Polyclonal, rabbit	PFA	1/100	Fisher scientific	10332473

## Data Availability

The datasets presented in this article are not readily available because the requester needs to be qualified by the authors beforehand. Requests to access the datasets should be directed to nc@neuro-sys.com.

## Supplementary Materials

The supporting information can be downloaded at: www.mdpi.com/article/10.3390/ijms23179864/s1. Reference [50] is cited in the supplementary materials.

## Abbreviations

6-Hydroxydopamine	6-OHDA
α-synuclein	α-syn
Apoptotis Inducing Factor: Aif Antibody	Ab
Cellular Prion Protein	PrPc
Conduritol B Epoxide	CBE
Cytochrome C	CytC
Dopamine	DA
Dopamine Transporter	DAT
Endoplasmic Reticulum	ER
Glucocerebrosidase	GCase
Reactive Oxygen Species	ROS
Paraformaldehyde	PFA
Phosphate Buffer Saline	PBS
Pre-Formed Fibrils	PFF
Soluble Nsf Attachment Protein	SNAP
Soluble Nsf Attachment Protein Receptor	SNARE
Substantia Nigra Pars Compacta	SNpc
Tyrosine Hydroxylase	TH
