# Supplementary material for: Alpha-Synuclein: The Spark That Flames Dopaminergic Neurons, In Vitro and In Vivo Evidence
Source: Int J Mol Sci. 2022 Aug 30;23(17):9864. doi: 10.3390/ijms23179864 (PMC9456396; doi:10.3390/ijms23179864)
Supplement: Supplementary file 1 [file ijms-23-09864-s001.zip › ijms-1832679-supplementary.pdf]

Supplementary

Materials and Methods

Alpha-synuclein preparation

Human α–syn peptide (stock at 69 µM in water) will be reconstituted at 50µM in NaCl, 0.9% (final concentration) and will be incubated at +37 °C for 3 days in dark and then frozen between each use. Of note, to reconstitute the peptide, a solution of NaCl at 1.24 % will be needed in order to have a final concentration at 0.9 % after dilution. The preparation includes monomeric form (20 %), small oligomers (from 25-100 kDa; 50 %) and large oligomers/fibrils (higher than 100 kDa; 30%)

GBA activity in primary culture of DA neurons, injured with CBE

Rat dopaminergic neurons were cultured as described in the main manuscript. On day 6 of culture, CBE (20µM) was added to the culture medium and let for 48h. On day 8 of culture, cells were washed with PBS, collected and lysed with a buffer lysis (CelLyticMT reagent, 1 % of Protease inhibitor). For each well, the quantity of protein will be determined using the micro kit BCA (Pierce). Enzymatic assay is based on the protocol described in Bouscary et al. 2019[1]. Briefly, the assay consisted in quantifying fluorescence coming from the degradation of 4-methylumbelliferone addition (4-MU, Sigma-Aldrich) by GBA in an acidic environment (pH 4.6) and in presence of detergent (Triton).

GBA activity in the whole brain

Mice were deeply anesthetized with ketamine chloralhydrate (120 mg/kg) and xylazine (16 mg/kg) and perfused with cold PBS (20mL). The two hemispheres were separated. The right one was snap frozen and kept at -80°C. Half brains were then lysed with potassium buffer (ph=7) and centrifuged for 15min (12 000 rpm). The supernatant was collected and stocked at -80°C. GBA enzymatic activity was assessed using the protocol described in Bouscary et al., 2019 [1].

Results

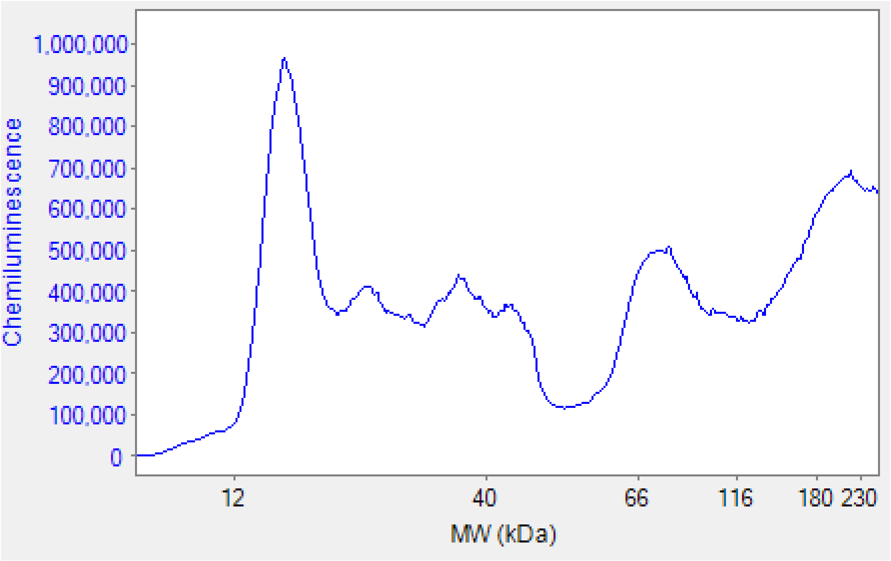

Figure S1 – Characterization of α-syn species by automated protein analysis. Electropherogram generated by WES apparatus (Protein simple) allowing the quantification of each entity of alpha-synuclein

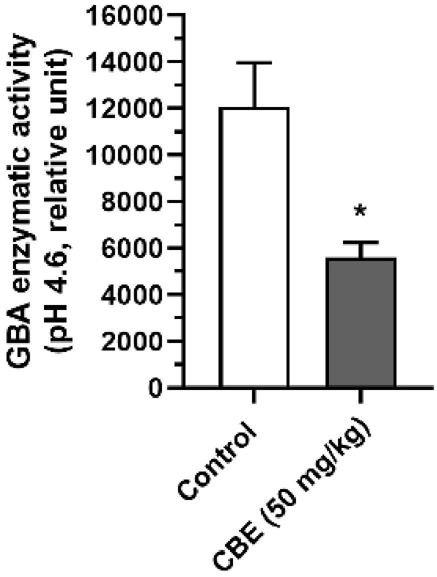

Figure S2 – Effects CBE on GBA activity in mice brain. All values are expressed as mean ± SEM; *, $p<0.05$ with non-parametric Mann-Whitney test.

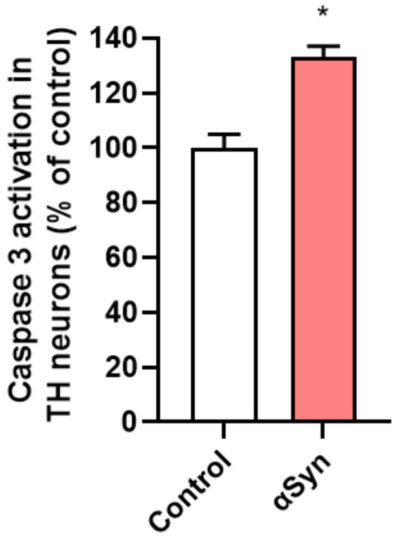

Figure S3— Effects of α-syn incubation on the activation of caspase 3. Area of cleaved/active caspase 3 in dopaminergic neurons (control = 25 µm2 ± 1.4) in TH(+) after intoxication with α-syn**.** All values are expressed as mean ± SEM; *, p<0.05 with student t test.

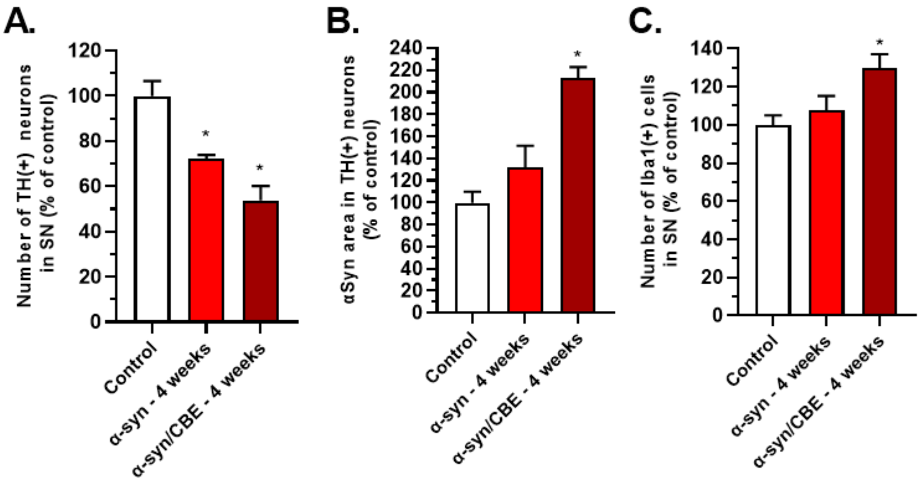

Figure S4 – Effects of α-syn and α-syn/CBE in SNpc of aged mice. Total number of TH neurons (control = 96; ±6,6) (A), area of cytoplasmic α-syn in TH neurons (control = 453,48 µm^2; ±9,9) (B) and microglial activation (control = 33,4; ±5,1) (C) were studied. All values are expressed as mean ± SEM; *, p<0.05 with One way ANOVA followed by Fisher's test

References

1. Bouscary A.; Quessada, C.; Mosbach, A.; Callizot, N.; Spedding, M.; Loeffler, J.P.; Henriques, A. Ambroxol Hydrochloride Improves Motor Functions and Extends Survival in a Mouse Model of Familial Amyotrophic Lateral Sclerosis. *Front. Pharmacol*. **2019**, *10*, 883. https://doi.org/10.3389/fphar.2019.00883.
